# Mitochondrial Dynamics: Biogenesis, Fission, Fusion, and Mitophagy in the Regulation of Stem Cell Behaviors

**DOI:** 10.1155/2019/9757201

**Published:** 2019-04-07

**Authors:** Wenyan Fu, Yang Liu, Hang Yin

**Affiliations:** ^1^Center for Molecular Medicine, The University of Georgia, GA 30602, USA; ^2^Department of Biochemistry and Molecular Biology, The University of Georgia, GA 30602, USA

## Abstract

Stem cells have the unique capacity to differentiate into many cell types during embryonic development and postnatal growth. Through coordinated cellular behaviors (self-renewal, proliferation, and differentiation), stem cells are also pivotal to the homeostasis, repair, and regeneration of many adult tissues/organs and thus of great importance in regenerative medicine. Emerging evidence indicates that mitochondria are actively involved in the regulation of stem cell behaviors. Mitochondria undergo specific dynamics (biogenesis, fission, fusion, and mitophagy) during stem cell self-renewal, proliferation, and differentiation. The alteration of mitochondrial dynamics, fine-tuned by stem cell niche factors and stress signaling, has considerable impacts on stem cell behaviors. Here, we summarize the recent research progress on (1) how mitochondrial dynamics controls stem cell behaviors, (2) intrinsic and extrinsic factors that regulate mitochondrial dynamics, and (3) pharmacological regulators of mitochondrial dynamics and their therapeutic potential. This review emphasizes the metabolic control of stemness and differentiation and may shed light on potential new applications in stem cell-based therapy.

## 1. Introduction

Embryonic stem cells (ESCs) have the pluripotent potential to generate all adult cell types. Adult stem cells instead are multipotent or unipotent and only give rise to limited numbers of cell types. By definition, stem cells must reproduce themselves, a process called self-renewal. Stem cell self-renewal is of great importance to the long-term maintenance of stem cell populations and the transient expansion of stem cells during development and tissue regeneration. Stem cell can self-renew through asymmetrical or symmetrical cell divisions. Through asymmetric cell division, a stem cell gives rise to a daughter stem cell and a daughter progenitor cell. The latter usually has limited lineage potential or progresses closer to the terminal differentiation. Progenitor cells can further differentiate into mature cell types, but by definition, progenitor cells lose their long-term self-renewing potential. Under the homeostatic condition, stem cells keep a delicate balance between self-renewal and differentiation through various intrinsic and extrinsic mechanisms [1]. Defects in stem cell self-renewal lead to their depletion and senescence, eventually result in developmental defects, failed tissue homeostasis, impaired tissue regeneration, and cancer [2, 3].

Differentiated somatic cells can be reprogrammed to induced pluripotent stem cells (iPSCs) by modulating specific transcription factors and/or signaling pathways. The ability to reprogram patient-specific cells into iPSCs offers therapeutic strategies in regenerative medicine for many congenital and acquired human diseases. iPSCs possess many characteristics similar to ESCs and adult stem cells, indicative of conserved mechanisms in regulating stem cell behaviors. Elucidating mechanisms that control stem cell behaviors have great significance in adult stem cell/iPSC-based regenerative medicine.

Mitochondria are the powerhouse of cells. Besides energy generation, mitochondria also participate in calcium signaling, redox homeostasis, differentiation, proliferation, and apoptosis. Mitochondria are quite dynamic organelles—they continuously undergo biogenesis, fission, fusion, mitophagy, and motility. Mitochondrial dynamics differs in different types of cells and meets the specific functional needs of the cell. Mitochondrial fission (mito-fission) allocates mitochondrial contents during cell division, generates heterogeneity, and aids in eradicating damaged mitochondria. Mitochondrial fusion (mito-fusion) enables mitochondrial content exchange and calcium and ROS buffering, promoting overall mitochondrial function. Coordinated biogenesis and mitophagy ensure sustainable mitochondrial functions. Overall, mitochondrial dynamics assists cells in meeting the needs for cellular energy during proliferation, differentiation, and apoptosis. In stem cells, the dynamics of mitochondria tightly connects to stem cell behaviors. Disrupting or modulating mitochondrial dynamics can have profound impacts on stem cell behaviors. Addressing how stem cell behaviors interplay with mitochondrial dynamics sheds light on the fascinating stem cell biology and also holds a promise to improve clinical applications of stem cells for regenerative medicine.

## 2. Mitochondrial Dynamics in Stem Cells and Differentiated Cells

**Figure 1:**
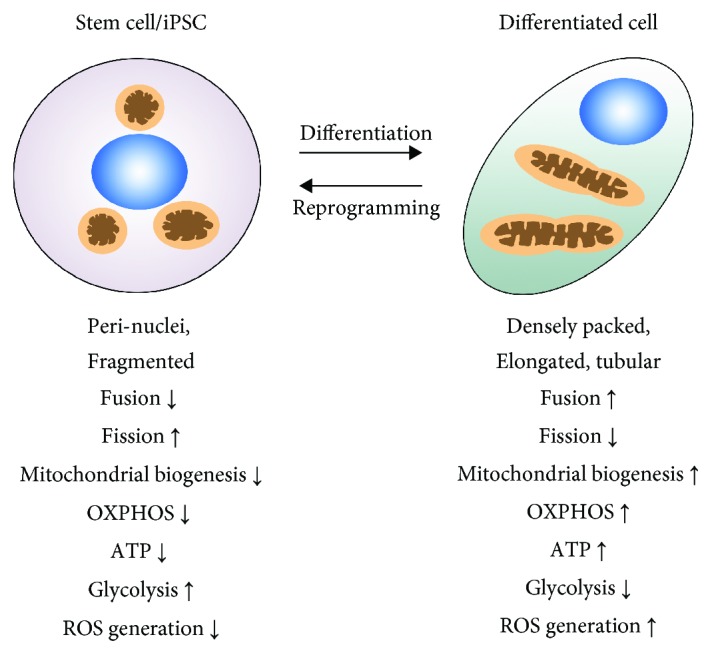
A simplified common scheme of mitochondrial dynamics in stem cells and differentiated cells. In most types of stem cells and reprogrammed iPSCs, mitochondria are usually localized in the nuclear periphery and characterized by sphere, fragmented, and punctate morphologies with fewer cristae (immature morphology). Correspondingly, mito-fission is high whereas mitochondrial biogenesis is low, which maintains low mitochondrial mass. Stem cells generally rely on glycolysis as the major energy source and have low levels of ATP, OXPHOS, and ROS levels. In differentiated cells, mitochondria change to more enlarged and elongated tubular morphology. Correspondingly, mito-fusion and biogenesis increase with the accumulation of mitochondria. Comparably, differentiated cells have higher ATP, ROS, and OXPHOS levels.

Mitochondrial dynamics differs between stem cells and differentiated cells (Figure 1). In stem cells, mitochondria are generally characterized as perinuclear-localized, in sphere, fragmented, and punctate shapes, and with fewer cristae. It is generally believed that mitochondria in stem cells are in an immature state, in which OXPHOS, ATP, and ROS levels are low. This state of mitochondria matches the overall function of stem cells—in a simplified point of view, stem cells serve to preserve the nuclear genome, epigenome, and mitochondrial genomes for differentiated cells. Thus, an immature state of mitochondria helps stem cells protect against ROS-induced genotoxicity, which would lead to more widespread and disastrous consequences in stem cells than in differentiated cells. Upon differentiation to terminal cell types, mitochondrial content increases, which is concomitant with the change of mitochondrial morphology—the appearance of enlarged, elongated, and tubular shapes. In differentiated cells, mitochondria are densely packed, and some are highly branched and distributed throughout the cytoplasm. Along with the maturation, mitochondrial ATP, OXPHOS, and ROS levels also increase in differentiated cells. The switch of cellular metabolism from glycolytic to oxidative types has been observed in the differentiation processes of many stem cell populations [4–7].

Stem cells and terminally differentiated cells also possess different mitochondrial dynamics, which is associated with the changes in morphology and metabolism during differentiation. At the transcriptional level, elevated mRNA of mito-fission gene *Drp1* is detected in ESCs and iPSCs comparing to differentiated cells. DRP1 protein and its active form phosphorylated DRP1 (p-DRP1 Ser 616) accumulate more in ESCs or iPSCs than in differentiated cells [8–11]. On the other hand, differentiated cells have increased abundance of mito-fusion genes *Mfn1* and *Mfn2* mRNAs [10] as well as elevated protein levels of MFN1, MFN2, and OPA1 in differentiated cells [9, 12]. The above correlations indicate that the differentiation processes of ESCs/iPSCs are concomitant with a shift from mito-fission in ESCs and iPSCs to mito-fusion in differentiated cells.

Besides mitochondrial fission and fusion, the expression levels of genes that are crucial for mitochondrial biogenesis (e.g., PGC-1*α*, PGC-1*β*, TFAM, and NRF1) also increase during the early differentiation of stem cells. This is accompanied with increased mitochondrial proteins and elevated mitochondrial mass [13–15]. Thus, the differentiation process is also associated with increased mitochondrial biogenesis.

## 3. Mitochondrial Dynamics Controls Stem Cell Behaviors

### 3.1. Mito-Fission

**Figure 2:**
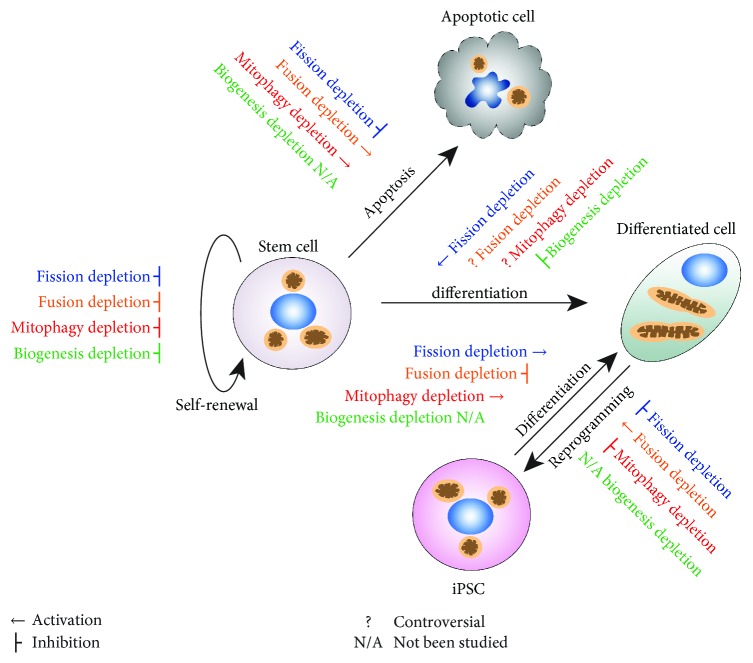
Modulating mitochondrial dynamics impacts on stem cell behaviors. Blockades of mitochondrial dynamics, fission (blue), fusion (orange), mitophagy (red), and biogenesis (green), affect stem cell differentiation, self-renewal, apoptosis, differentiation, and reprogramming. Downregulation of mito-fission usually leads to impaired self-renewal and the loss of stemness in stem cells, while increasing differentiation. Stem cells are often protected from apoptosis. Fission blockade also decreases the reprogramming efficiency. Downregulation of mito-fusion impairs stem cell self-renewal and may have diverse effects on stem cell differentiation. In general, mito-fusion protects stem cells from apoptosis, and the mito-fusion blockade often results in increased vulnerability to stress. Downregulation of mito-fusion improves the reprogramming efficiency. The blockade of mitophagy also impairs stem cell self-renewal as well as decreases reprogramming efficiency. The function of mitophagy in stem cell differentiation has not been understood clearly enough and may be stem cell type-specific and lineage-specific. Mitochondrial biogenesis is generally pivotal for stem cell maintenance. Downregulation of biogenesis impairs stem cell self-renewal and differentiation. More detailed information on stem cell behaviors and their regulation by mitochondrial dynamics are listed in Table 1.

Mitochondrial dynamics not only indicates undifferentiated vs. differentiated states of stem cells but also, reversely, modulates stem cell behaviors (Figure 2 and Table 1). The fragmented morphology of mitochondria in stem cells leads to an intriguing question—is mito-fission essential for the stemness? As Drp1 plays a critical role in mito-fission, some recent studies sought to answer the question by genetically knocking out or knocking down Drp1 or pharmacologically inhibiting Drp1 with its specific inhibitor, mitochondrial division inhibitor 1 (mDivi-1). In human iPSCs (hiPSCs), both Drp1 knockout and Drp1 inhibitor mDivi-1 treatment promote hiPSCs to differentiate into cardiomyocytes with augmented cardiac-specific gene expression. In addition, Drp1 downregulation in hiPSCs also elicits the metabolic switch from glycolysis (featured in stem cells) to OXPHOS (features in differentiated cells) [10]. iPSCs treated with mDivi-1 lose their typical morphology and adopt shapes resembling differentiated cells instead. mDivi-1-treated iPSCs also have reduced alkaline phosphatase (AP) staining [16], in line with the loss of stemness upon Drp1 downregulation. A similar effect of mito-fission on stemness is also observed in the cancer stem cells. In nasopharyngeal carcinoma cells (NPC), stem cell markers Oct4 and ABCG2 diminish when Drp1 activation (p-Drp1 Ser 616) is downregulated by Cox2 blockade, indicating the loss of stemness [17].

Drp1-dependent mito-fission is also associated with stem cell asymmetric division. Katajisto et al. reported that mitochondria are asymmetrically divided into daughter cells during stem cell division—the daughter cell that receives more young mitochondria becomes the self-renewed stem cell [18]. The asymmetric division of young and old mitochondria depends on Drp1-mediated mito-fission. Interestingly, Drp1 inhibition by mDivi-1 results in the random allocation of young and old mitochondria during stem cell division and impaired self-renewal and stemness [18].

Besides the maintenance of stemness, mito-fission also has a critical function in the progress of reprogramming—the *de novo* establishment of stemness. Knocking down mito-fission mediators, Drp1, Mid51, and Gdap1, markedly decreases the reprogramming efficiency as evidenced by a fewer number of alkaline phosphatase- (AP-) positive adherent colonies during reprogramming [11, 19].

Reprogramming is a stepwise process. Differentiated cells must overcome several barriers to obtain pluripotency. As stem cells and differentiated cells have distinct mitochondrial characteristics, the remodeling of mitochondria is conceivably one of the obstacles for reprogramming. It is generally accepted that mito-fission is induced during reprogramming [8, 11]. However, whether increasing mito-fission can increase reprogramming efficiency remains controversial. A possible explanation for the conflicting observations may lie in side effects (e.g., ROS production, apoptosis, and mitochondria integrity impairment) caused by excessive fission. Many pathways and factors have been implicated in activating mito-fission. Of note, fatty acid synthesis promotes mito-fission and also improves the reprogramming into hiPSCs [20]. In this scenario, fatty acid synthesis seemingly promotes fission in a mild and healthy level, at which mitochondria remain in a good condition for reprogramming. In contrast, excessive fission apparently impairs stemness of embryonic stem cells (ESCs) [21]. Excessive fission increases intracellular Ca^2+^ level and CaMKII activity, leading to the degradation of *β*-catenin, a critical factor for pluripotency maintenance [21]. Growth factor erv1-like (Gfer) represses Drp1 expression and mito-fission. Gfer downregulation in ESCs augments Drp1-dependent mito-fission yet results in the loss of stemness [22]. In this scenario, the loss of stemness seems to attribute to apoptosis, supporting the above notion that only an appropriate level of mito-fission promotes the establishment of stemness in reprogramming. Besides apoptosis, excessive mito-fission also leads to the abnormal accumulation of ROS [23–26] and causes the loss of self-renewal capacity in some stem cell populations [27–30]. It is noteworthy that a moderate level of ROS is necessary for the maintenance of self-renewal in some types of stem cells [31, 32]. Thus, balancing the impacts of mito-fission on mitochondrial functions (e.g., bioenergetics, ROS generation, and apoptosis) is pivotal for the maintenance and establishment of stemness.

Notably, mito-fission is elevated under stress conditions [33–35]. This is mostly studied in nonstem cells. In physiological conditions with mild stress, mito-fission is associated with prosurvival mitophagy to clear defective mitochondria [36, 37]. However, under extreme stress conditions, the mitochondrial network is fragmented due to extensive mito-fission. Drp1- and Fis1-mediated mito-fission contributes to apoptosis [38–40]. Inhibiting Drp1 activity prevents the loss of mitochondrial membrane potential and the release of cytochrome *c* in Hela and COS7 cells and hence protects against apoptosis [39, 40]. Drp1 activity is controlled by its phosphorylation at serine 656 residue (p-Drp1 Ser 656). Sympathetic activity activates cAMP-dependent protein kinase (PKA), which phosphorylates Drp1 at serine 656 and consequently inhibits Drp1 activity in PC12 cells. Reversely, calcium mobilization and the activation of calcineurin phosphatase lead to dephosphorylation of this site on Drp1 and hence apoptosis [38]. In Hela cells, the downregulation of mitochondrial fission 1 (Fis1) robustly inhibits cell death [40]. Back to the stem cell context, Drp1 inhibitor mDivi-1 blocks Drp1 translocation from the cytosol to mitochondria and protects rat hippocampal neural stem cells from palmitate-induced apoptosis and cell death [41]. Thus, inhibiting mito-fission may hold potential in protecting stem cells from apoptosis under pathological stress conditions.

### 3.2. Mito-Fusion

Mito-fusion enables content exchange between individual mitochondrion as well as between mitochondria and nucleus. Mito-fusion requires the coordination of multiple interacting factors. The fusion of mitochondrial outer membrane is mediated by Mfn1 and Mfn2. Inner membrane fusion requires long-form OPA1. All the mediators are associated with fusion-mediated regulation of stem cell behaviors (Figure 2 and Table 1).

Mito-fusion is necessary for stem cell differentiation. In most differentiated somatic cells, mitochondria are in tubular and network structure. In *Drosophila* intestinal stem cells (ISCs), defective mito-fusion due to OPA1 knockdown impairs stem cell differentiation [42]. Under a prodifferentiation condition, OPA1-/- ISCs do not express differentiation-specific markers but instead show the characteristics of stem cells [42]. The gene trapping of mito-fusion protein Mfn2 or OPA1 in ESCs exhibits the same phenotype—the differentiation of ESCs to cardiomyocytes is blunted [12]. Factors regulating mito-fusion are also involved in the fate determination of stem cells. Mitochondrial carrier homolog 2 (MTCH2) is a regulator of mito-fusion, metabolism, and apoptosis. In MTCH2-/- ESCs, mitochondria fail to elongate and the stem cells have a delay in exiting the naïve pluripotency stage upon differentiation stimulation. Interestingly, Mfn2 overexpression or a dominant negative form of Drp1 rescues mito-fusion in MTCH2-/- ESCs and drives the stem cells to exit the naïve state and enter the prime state [43].

Mito-fusion is apparently essential for iPSC differentiation as well. In neurogenic differentiation of hiPSCs, Mfn2 knockdown results in deficits in neurogenesis and synapse formation [44]. In contrast, overexpression of Mfn2 in hiPSCs can promote the differentiation and maturation of neurons [44].

Although plenty of evidence indicates that the blockade of mito-fusion impedes stem cell differentiation, this notion cannot be generalized to all types of stem cells or all cell fate lineages. For example, in neural stem cells (NSCs), mitochondria are in the tubular structure instead of fragmented [30]. It would be expected that mito-fusion may differently impact on NSC differentiation. Indeed, the knockout of mito-fusion genes reduces the self-renewing capacity of neural stem cells due to ROS accumulation and NRF-2-mediated retrograde signaling [30]. Murine mesenchymal stem cells (MSCs) represent another example for differential requirements of mito-fusion in lineage differentiation. MSCs are multipotent stem cells that can differentiate into adipocytes, osteocytes, and chondrocytes. During adipogenic and osteogenic differentiation, the expression of mito-fusion factors increases and mitochondria fuse and elongate. However, chondrogenic differentiation is accompanied with fragmented mitochondria and increased expression of mito-fission factors [45]. With Mfn2 downregulation in MSCs, the differentiation into adipogenic and osteogenic lineages fails, whereas chondrogenesis is abolished only when Drp1 is downregulated [45]. Intracellular ROS levels may contribute to the diverse effects of mito-fusion on stem cell differentiation. It has been observed that mitochondria adopt a fragmented structure and produce more ROS in fusion-deficient stem cells [30, 42]. ROS apparently have different effects on stem cell differentiation. For example, in mesenchymal stem cell (MSC) differentiation, a high level of ROS favors adipogenesis whereas a low ROS level prefers osteogenesis [46]. Multiple REDOX sensors (e.g., p38-MAPK, ERK1/2, and JNK) may mediate the diverse effects of ROS on stem cell differentiation. Cleary, our knowledge in the interplay between mito-fusion and stem cell differentiation is far from complete.

Only a limited number of studies have reported the interaction between mito-fusion and stem cell self-renewal. In NSCs, dampening mito-fusion by deleting OPA1 or Mfn1/2 impairs the self-renewing capacity, suggesting that mito-fusion is necessary for self-renewal [30]. On the other hand, mito-fusion seemingly facilitates stem cell self-renewal. Wu et al. reported that the epithelial-mesenchymal transition (EMT) of mammary stem cells induces mito-fusion through miR200c-PGC1*α*-Mfn1 pathway [47]. Mfn1 is required for PKC*ζ*-mediated NUMB phosphorylation and hence directs asymmetry division and self-renewal [47]. As to reprogramming, it has been reported that Mfn1/2 depletion promotes reprogramming and the maintenance of pluripotency. The downregulation of Mfn1/2 activates Ras-Raf and HIF-1*α* and facilitates the transition to glycolytic metabolism [9].

Opposite to mito-fission, which induces apoptosis and cell death, mito-fusion protects cells from apoptosis. COS7 cells with activated Mfn2 have an increase of the nucleotide exchange rate, and the cells are protected against free radical-induced depolarization. The underlying mechanism is shown—the activated Mfn2 interferes with BAX activation and cytochrome *c* release [48]. With the overexpression of rat Fzo1 (a counterpart of human Mfn proteins), Hela cells adopt an elongated mitochondrial structure and become protected from etoposide-induced cell death. Reversely, gene silencing of Fzo1 causes an increase of susceptibility to radical-induced cell death [49].

### 3.3. Mitophagy

Mitochondrial quality and integrity are essential for normal functions of mitochondria. Defective mitochondria can be cleared by mitophagy, which plays critical roles in stem cell maintenance (Figure 2 and Table 1). Multiple studies indicated that stem cell self-renewal relies on mitophagy. In hematopoietic stem cells (HSCs), Atg12 knockout blockades mitophagy and results in aberrant accumulation of mitochondria. The self-renewal and differentiation potential of HSCs are impaired by Atg12 knockout, which exacerbates during aging [50]. In Atg3 knockout ESCs, the accumulation of defective mitochondria is accompanied by elevated ROS production, leading to the impairment of self-renewal [51]. In human leukemia stem cells (LSCs), Fis1 (mitochondrial fission 1) depletion attenuates mitophagy, leading to cell cycle arrest and impaired self-renewal. It has been shown that AMPK activates Fis1-dependent mitophagy, and AMPK inhibition mimics the Fis1 depletion-induced mitophagy defect [52]. In Tie2^+^ HSCs, mitophagy is essential for self-renewing expansion [53]. It was shown that the activation of PPAR-fatty acid oxidation pathway promotes HSC self-renewing expansion by recruiting Parkin to mitochondria. Silencing Pink1 or Parkin not only abrogates the self-renewal but also inhibits the maintenance of Tie2^+^ HSCs [53].

Likewise, mitophagy is necessary for reprogramming. Loss of Pink1-dependent mitophagy dampens reprogramming efficiency [54]. Similar negative effect on reprogramming was also observed in response to Atg3 knockout-induced mitophagy defect [51].

Both stem cell self-renewal and iPSC reprogramming require mitochondria in high quality and a low level of ROS. Excessive ROS have been detected in mitophagy-defective stem cells [51, 55, 56]. Given the detrimental effects of ROS on stem cell self-renewal, it is reasonable to conceive that mitophagy has a pivotal role in protecting stem cells from the loss of self-renewal and maintenance.

The function of mitophagy in stem cell differentiation may vary in different types of stem cells and differ at stages in the differentiation process. Mitophagy defect in Atg12 knockout HSCs leads to differentiation [50]. Atg12-/- HSCs express higher levels of premyeloid markers and form unipotent mature colonies. Although Atg12-/- HSCs have elevated ROS levels, the prodifferentiation effect is likely not driven by ROS accumulation because a ROS scavenger NAC does not abolish the differentiation [50]. Similarly, mitophagy deficiency induced by Pink1 knockout in iPSCs promotes differentiation. Pink1-/- iPSCs have strong tendency to spontaneously differentiate into heterogeneous cell types [54]. However, it should be kept in mind that abnormal differentiation could occur under mitophagy deficiency, as exampled by delayed expression of certain endoderm and mesoderm markers during the differentiation of Atg3-/- ESCs [51]. On the other hand, mitophagy deficiency may impede stem cell differentiation in some types of stem cells. For example, the loss of Pink1 in NSCs leads to retarded differentiation towards mature neurons with an unknown mechanism [57]. During C2C12 myoblast differentiation, mitophagy is induced in the early stage of myogenesis to clear preexisting mitochondria and make way for newly generated OXPHOS-competent mitochondria from a burst of mitochondrial biogenesis [58]. In this scenario, mitophagy blockade impairs myogenic differentiation.

As a mitochondrial quality control, mitophagy acts to protect cells from apoptosis. In chlorpyrifos-induced apoptosis of SH-SY5Y cells, Pink1/Parkin-mediated mitophagy is increased and Parkin knockdown drastically increases apoptosis. On the other hand, Parkin overexpression alleviates apoptosis [59]. Similarly, mitophagy protects human vascular smooth muscle cells from atherogenic lipid-induced apoptosis as evidenced by extensive apoptosis upon Pink1/Parkin silencing [60]. Bnip3 is another mitophagy-related protein that protects against apoptosis. The phosphorylation of Bnip3 drives prosurvival mitophagy to protect HL-1 cardiac cells from apoptosis [61]. The phosphorylation of serine residues 17 and 24 flanking Bnip3 LIR (LC3 binding region) promotes its binding to LC3B, which signals mitochondria for lysosomal degradation prior to cytochrome *c* release-induced apoptosis [61]. In NSCs, Pink1 knockout leads to an increase of apoptosis in the absence of stress [57].

### 3.4. Mitochondrial Biogenesis

In both ESCs and iPSCs, mitochondrial biogenesis is concomitant with differentiation (Figure 2 and Table 1) [13, 14]. Peroxisome proliferator-activated receptor gamma coactivators (PGC-1) play pivotal roles in mitochondrial biogenesis. The genetic deletion of both PGC-1*α* and PGC-1*β* in brown adipocyte progenitors drastically abolishes the differentiation into brown adipocytes [62]. On the other hand, the upregulation of mitochondrial biogenesis also facilitates differentiation [63]. It has been reported that Wnt signaling promotes osteoblastic differentiation of murine mesenchymal C3H10T1/2 cells in a mitochondrial biogenesis-dependent manner. Wnt activation induces biogenesis and augments mitochondrial ATP and ROS production. The suppression of mitochondrial biogenesis with AZT abrogates the differentiation upon Wnt activation. Consistent with the observation, stimulating mitochondrial biogenesis with TFAM further increases the differentiation.

Whether mitochondrial biogenesis directly impacts on stemness remains largely elusive. It is generally accepted that mitochondrial biogenesis maintains at a low level in stem cells, resulting in few mitochondria and a low level of ROS. The low level of mitochondrial biogenesis seems to be necessary for the stemness and quiescence. First, this note is supported by observations from HSCs in SDF-1/CXCL12 transgene mice with constitutive active CXCR4 pathway [64]. In these mice, mitochondrial biogenesis in HSCs is upregulated and the HSCs express increased CD34, indicative of a loss of Lin(-)/Sca1(+)/c-Kit(+) primitive state and long-term repopulating potential. In another study, mTOR activation, as a result of Tuberous Sclerosis Complex (TSC) knockout, increases mitochondrial biogenesis and ROS production in HSCs [65]. The quiescence in these HSCs is disrupted, which can be rescued by a ROS scavenger NAC. Although both studies reveal an intriguing correlation between an increase of mitochondrial biogenesis and the loss of stemness and stem cell quiescence, it remains unclear whether mitochondrial biogenesis is the direct cause of the above effects.

Downregulation of mitochondrial biogenesis via genetic approaches is not easy since such manipulations are always associated with cell death. Pharmacological inhibition of mitochondrial biogenesis may also affect cell survival. For example, XCT790 is a specific inhibitor of ERR*α*-PGC-1 signaling pathway and acts to inhibit mitochondrial biogenesis [66]. Treating cancer stem-like cells with XCT790 lowers cell viability and suppresses Wnt, STAT3, and TGF-*β* prosurvival pathways. The cell death may be due to low-energy stress caused by reduced mitochondrial mass and function, as a supplement of acetyl-L-carnitine can rescue cell death.

## 4. Mitochondrial Dynamics Is Regulated under Stress

**Figure 3:**
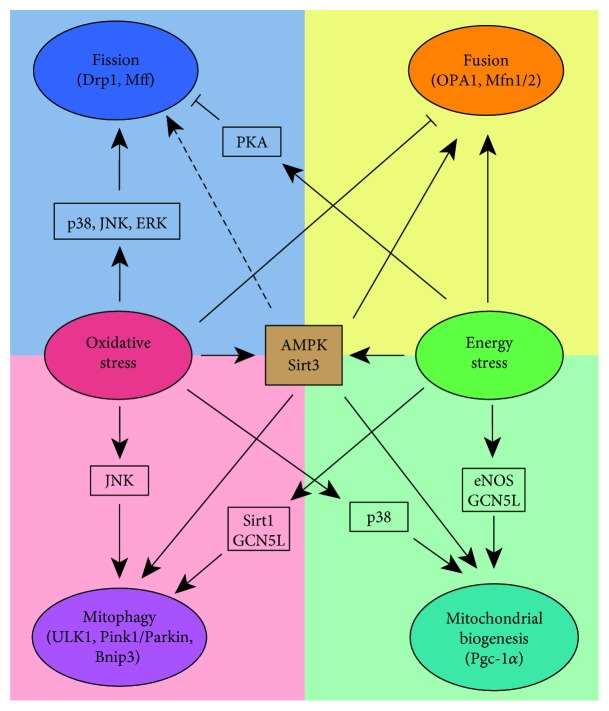
Mitochondrial dynamics is regulated through multiple pathways. Oxidative stress and energy stress have distinct impacts on mito-fission (blue), mito-fusion (orange), mitophagy (pink), and mito-biogenesis (green) via distinct signaling pathways. The dash line denotes that the results from multiple studies are conflicting.

Many (but not all) types of adult stem cells with long-term self-renewing capabilities exist in a quiescent or slow-proliferating state. Stem cells in this state are often exposed to relatively low levels of oxygen and growth factors from their niches. As discussed before, mitochondrial dynamics has profound impacts on stem cell behaviors. Here, we review some recent findings on stress-induced alterations of mitochondrial dynamics, which conceivably also affect stem cell behaviors (Figure 3).

### 4.1. Oxidative Stress

ETC activities in mitochondria is a major source of intracellular ROS. ROS is neutralized by various antioxidant defense systems. Oxidative stress occurs when an excessive amount of ROS accumulates. Accumulating evidence suggests that ROS regulate mitochondrial dynamics.

In stem cells and progenitor cells, oxidative stress promotes mito-fission. Oxidative stress induced directly by hydrogen peroxide (H_2_O_2_) treatment results in mitochondrial fragmentation in myoblasts. Drp1-specific inhibitor, mDivi1, attenuates mitochondrial fragmentation, indicating the fragmentation is Drp1-dependent [33]. Hypoxia promotes mito-fission in stem cells [34, 67, 68]. In periodontal ligament stem cells, CoCl_2_ treatment, a condition mimicking hypoxia (pseudohypoxia), results in ROS-mediated mito-fission and apoptosis, which can be rescued by NAC [67]. Energy overloading can also induce oxidative stress and promote mito-fission [20, 69]. Prolonged exposure to saturated free fatty acids (e.g., palmitate) is cytotoxic to neural stem cells, which can be prevented by the Drp1 inhibitor mDivi-1 [41].

Intriguingly, ROS can function as signaling molecules to regulate stem cell behaviors via REDOX sensors. It is well known that ROS can activate many MAPKs, including p38, ERK1/2, and JNK [70]. These MAPKs, functioning as REDOX sensors, have diverse functions in stem cells [11, 28, 71, 72]. One particular function is to regulate oxidative stress-induced mito-fission. The activation of ERK1/2 further phosphorylates Drp1 to promote mito-fission, which results in stem cell proliferation or reprogramming [11, 73]. Succinate induces ROS and promotes human mesenchymal stem cell (hMSC) migration. The activation of PKC upon succinate treatment activates p38 MAPK, which leads to Drp1 translocation onto the mitochondrial outer membrane for fission.

ROS have opposite effects on mito-fusion. Oxidative stress disrupts mito-fusion and leads to fragmented mitochondrial morphology. In osteosarcoma and cardiomyoblasts, oxidative stress (induced by H_2_O_2_ exposure) decreases the active OPA1 isoform [74]. Consistently, in fibroblasts, H_2_O_2_ treatment results in polyubiquitination-mediated Mfn1/2 degradation [75].

ROS-induced mito-fission is vital for mitophagy. Aberrant ROS accumulation in mitochondria causes mitochondrial dysfunction and the defective mitochondria need to be cleared by mitophagy. Pink1 and Parkin promote Mfn1/2 ubiquitination and increase mito-fission-dependent mitophagy to clear ROS-overloaded mitochondria [75]. Moreover, many studies have demonstrated that augmented ROS is a trigger for mitophagy [76–79]. REDOX sensor JNK participates in ROS-induced mitophagy via mitophagy-related protein Bnip3 [80]. It is found that Bnip3 expression in cardiomyocytes is correlated to JNK activity. Prolonged JNK activation overrides the inhibitory effect of AKT on FOXO3, resulting in elevated FOXO3 activity and the expression of its target Bnip3. In this scenario, mitophagy is induced by JNK activation [80].

ROS also promote mitochondrial biogenesis. ROS are found to upregulate PGC-1*α* activity in various cellular contexts [81–84]. Many signaling pathways have been shown to regulate PGC-1*α* in response to oxidative stress. One of these pathways is p38 MAPK signaling, which activates PGC-1*α* [85, 86]. In C2C12 myoblasts, p38 MAPK phosphorylates PGC-1*α* (at residues threonine 262, serine 265, and threonine 298) and stabilizes PGC-1*α* protein [86]. Another example is the induction of Sirt3, a deacetylase enriched in mitochondria, by oxidative stress. Sirt3 overexpression in neurons protects against oxidative stress-induced neuronal injury via orchestrating Ca^2+^ homeostasis and mitochondria biogenesis [87]. In human umbilical endothelial cells, H_2_O_2_ activates Sirt3 to deacetylate FOXO3, thus increasing PGC-1*α* and TFAM expression [88].

### 4.2. Energy Stress

Mitochondria elongate upon energy deprivation, which is mediated by the downregulation of active Drp1 (p-Drp1 Ser 616) and redirection of Drp1 from mitochondria [89]. cAMP-dependent protein kinase (PKA) signaling pathway controls cell growth in response to nutrient deprivation [38, 90]. PKA is activated upon energy depletion. Active PKA phosphorylates Drp1 (p-Drp1 Ser 637) and inactivates Drp1, which inhibits mito-fission in Hela cells [90]. It remains unknown whether PKA is responsible for p-Drp1 Ser 616 increase under starvation [91]. AMPK, another sensor of energy stress, is induced by both energy deprivation and ROS [70, 92–94]. Numerous studies have established tight connections between AMPK and mitochondrial dynamics. In endothelial cells, pharmacological activation of AMPK with AICAR prevents mito-fission by inactivating Drp1 [95]. On the other hand, the activation of AMPK with AICAR in U2OS cells is sufficient to promote mito-fission in the absence of mitochondrial stress [35]. It has been reported that active AMPK phosphorylates Mff at Ser 155 and Ser 172 residues, which is required for Drp1 recruitment to mitochondria during fission [35]. The function of AMPK on mitochondrial fission may depend on the cellular need. Upon extreme stress conditions, AMPK-mediated fission might be a dominant effect to facilitate mitochondria clearance. When damaged mitochondria are eliminated and stress is reduced, AMPK may help cells restore mitochondrial function by promoting fusion instead of fission.

In contrast to mito-fission, starvation induces mito-fusion via upregulating Mfn1 [89]. It has been postulated that the tubular mitochondrial structure in mouse embryonic fibroblasts under energy stress prevents mitochondria from autophagy-induced mitophagy. Maintaining mitochondrial mass or even increasing mitochondrial mass upon starvation permits mitochondria to maximize the energy supply for the whole cell [89]. AMPK is also a key regulator for mito-fusion at least in the aging context. In *C. elegans*, APMK and dietary restriction protect body-wall muscle cells from aging by maintaining mitochondria in a fusion state [96]. In addition, the activation of AMPK with its activator AICAR can induce fusion in rat hepatocyte and protect the cells from drug-induced apoptosis [97]. Sirt3 also promotes mito-fusion. Sirt3 deacetylates OPA1 and increases its GTPase activity for mitochondrial fusion [98]. The Sirt3-dependent activation of OPA1 preserves the mitochondrial network and protects cardiomyocytes from doxorubicin-mediated cell death [98]. Mito-fusion conceivably can buffer stress conditions in mitochondria. The regulation of mito-fusion by Sirt3 echoes this function of mito-fusion as demonstrated by responses to calcium and ROS in neurons [87].

Energy stress increases NAD^+^/NADH ratio, which activates sirtuin family. Sirt1 has received much attention on its antiaging effects, which may be attributed to its indispensable role in maintaining mitochondria homeostasis. Sirt1 acts mainly in mitochondrial mitophagy [99–102]. The Sirt1-specific activator SRT1720 and NAM (promoting Sirt1 activity via increasing NAD^+^) decrease mitochondria content by facilitating mitophagy in human fibroblasts [99]. In line with this, Sirt1 inhibition is underlying impaired mitophagy in disease models. In DNA repair-deficient XPA mouse models, PARP1 activation blunts mitophagy through Sirt1 inhibition and causes mitochondrial dysfunction [100]. Sirt1 deacetylates autophagy-related proteins (LC3, Atg5, and Atg7), leading to phagophore maturation and mitophagy in mouse embryonic fibroblasts (MEF), HEK293, and Hela cells [101, 102]. Besides Sirt1, GCN5L and Sirt3, which specifically function as deacetylase in mitochondria, are also involved in mitophagy induced under energy deprivation. In MEF and HepG2 cells, long-term genetic depletion of GCN5L reduces mitochondrial mass via autophagy-induced mitophagy [103, 104], which is also reported to be Sirt3-dependent [104]. In MEF cells, GCN5L knockout has a positive effect on the expression and activity of transcriptional factor EB (TFEB), a master regulator of autophagy and its downstream targets [103]. Instead, Sirt3 deacetylates FOXO3 to increase the expression of mitophagy mediators (such as Bnip3, NIX, and LC3) [88]. AMPK also participates in mitophagy regulation under energy stress. As an energy sensor, AMPK phosphorylates ULK1 and hence connects energy sensing to mitophagy in MEFs [105] and exercise-induced mitophagy in mice [106]. As mentioned before, AMPK activates Fis1 in human leukemia stem cells (LSCs) to promote mitophagy and LSC stemness maintenance [52].

Mitochondrial biogenesis is often coincident with mitophagy. In GCN5L knockout MEFs, TFEB and PGC-1*α* are induced to promote mitochondrial biogenesis. The concurrent induction of mitophagy and biogenesis increases the mitochondrial turnover rate and ensures mitochondrial homeostasis [103]. Endothelial nitric oxide synthase (eNOS) is another factor that mediates energy deficiency-induced mitochondrial biogenesis. eNOS increases under caloric restriction and has an essential function in caloric restriction-induced mitochondrial biogenesis [107]. In the skeletal muscle, AMPK activation is also associated with mitochondrial biogenesis. In response to chronic energy deprivation, mitochondria undergo AMPK-dependent adaptive biogenesis [108, 109]. The treatment of *β*-GPA (mimicking chronic energy deprivation) activates AMPK and consequently results in mitochondria biogenesis in the muscle [110]. Mechanistically, AMPK may promote mitochondrial biogenesis by activating the nuclear respiratory factor-1 (NRF-1)/mTFA axis [111] or PGC-1*α* [110, 112]. By far, there is no direct evidence showing that the AMPK activator can induce mitochondrial biogenesis in stem cells.

## 5. Pharmacological Regulators of Mitochondrial Dynamics and the Potential Applications

### 5.1. Mito-Fission Inhibitors

As discussed above, inhibitors of mito-fission may protect stem cells from apoptosis and promote iPSC differentiation (Table 2). mDivi-1 is a widely used fission inhibitor, which inhibits the assembly of Drp1, and its GTPase activity meanwhile does not interfere with mito-fusion [113]. Several studies have reported that mDivi-1 protects stem cells from cell death. In type 2 diabetes, mDivi-1 treatment protects hippocampal neural stem cells from palmitate-induced apoptosis [41]. It has been discussed in this review that fission blockade promotes iPSC differentiation, which may have clinical potential in cardiac regeneration. In this regard, pharmacological inhibition of Drp1 with mDivi-1 increases mitochondrial respiration and promotes human iPSC differentiation into cardiac lineage-committed cells [10].

P110 is another mito-fission inhibitor, acting by blocking Drp1/Fis1 interaction [23]. P110 was first used as a protector for neuronal cells. In cultured neurons, P110 treatment prevents mitochondrial fragment and excessive ROS production, improves mitochondrial integrity and membrane potential, and protects the cells from stress-induced death [23]. Other studies have utilized P110 to inhibit mitochondrial fission for protecting the cell from stress- or injury-induced death, especially on cardiac disease models. In both *in vitro* and *in vivo* studies, P110 treatment improves acute infarction-induced cell death and prevents cardiac dysfunction [114].

Dynasore is a cell-permeable inhibitor of dynamin. Dynasore functions to noncompetitively inhibit the GTPase activity of Dynamin1, Dynamin2, and Drp1 and hence is used as a mito-fission inhibitor [115]. Similar to the other two inhibitors, dynasore protects the cardiomyocyte from ischemia/reperfusion injury *in vivo* [116]. Comparing to the other two inhibitors, dynasore has less specificity towards mito-fission inhibition.

In summary, although it has not been tested extensively, mito-fission inhibitors may have potential in stem cell-based regenerative medicine. Extra care should be taken to prevent the loss of stemness and stem cell homeostasis upon mito-fission inhibition.

### 5.2. Mito-Fusion Activators

Only limited numbers of mito-fusion regulators are currently available. One activator is fusion promotor M1, which was introduced in 2012. Mitochondrial fragmentation is prominent in 1-methyl-4-phenyl-pyridinium- (MPP+-) treated SH-SY5Y cells, which model the neuron cell death in Parkinson disease. In this model, mito-fusion promotor M1 treatment reduces cytochrome *c* release and protects cells from cell death [117]. Similarly, mito-fusion promotor M1 is also protective for in vitro Parkinson disease model induced by rotenone [118].

The other mito-fusion activator is leflunomide, a new chemical introduced in 2018. This activator was identified in a small-molecule compound screening for MFN1/MFN2-dependent mitochondrial elongation. Hela cells treated with leflunomide show elongated mitochondrial network and increased Mfn1/2 expression. Mechanistically, leflunomide seems to be effective via the inhibition of pyrimidine synthesis. Leflunomide can reduce doxorubicin-induced PARP and cleaved-caspase 3 activity in MEF cells and protect PC12 cells from apoptosis [119].

Although it has not been used in a study with stem cells, mito-fusion promotor M1 and leflunomide may have therapeutic potential in inducing stem cell/iPSC differentiation in clinical settings (Table 2).

### 5.3. Biogenesis Inhibitors

Pharmacological inhibition of mitochondria biogenesis causes cell death. However, it may serve as targeted mitochondrial therapies for cancer (Table 2). Some studies have tested mitochondrial biogenesis inhibitors on cancer stem cells, which tend to be chemoresistant. A characteristic of cancer stem cells is the high mitochondria content, which may allow therapeutic strategies to eradicate these cells by mitochondrial biogenesis inhibition [120]. XCT790 is a specific inhibitor of ERR*α*-PGC-1 signaling pathway and inhibits mitochondrial biogenesis. Treating cancer stem cells with XCT790 suppresses cell viability by reducing prosurvival pathways [66]. Human non-small-cell lung cancer cells treated with XCT790 display reduced mitochondrial mass as well as increased ROS level, which modulates p53 and Rb signaling pathway for cell cycle arrest [121]. However, the effect of CXT790 on normal cell has not been evaluated.

Azithromycin and doxycycline are FDA-approved antibiotics that inhibit mitochondrial biogenesis via inhibiting mitochondrial protein translation. These antibiotics inhibit tumor sphere formation in eight different types of cancer stem cells (breast, DCIS, ovarian, prostate, lung, pancreatic, melanoma, and glioblastoma) [122]. Of the two, doxycycline has lower toxicity to normal cells [123] and may also have a favorable anti-inflammatory effect [124].

## 6. Perspective

Accumulating evidence shows that mitochondrial dynamics delicately interplays with stem cell behaviors. Stem cell behaviors (self-renewal, maintenance, proliferation, cell fate determination, and differentiation) can be altered by modulating mitochondrial fission, fusion, mitophagy, and biogenesis. As an emerging field, there are many questions awaiting to be answered related to stem cells and mitochondrial dynamics. The recent advance in inhibitors and activators of mito-fission and mito-fusion may allow the modulation of mitochondrial dynamics in various stem cell models. Moreover, pathways that participate in stress-induced mitochondrial dynamics regulation and responsive to mitochondrial dynamics should be examined in stem cell populations.

## Figures and Tables

**Table 1:** A summary of the effects on stem cell behaviors upon modulating key factors in mitochondrial dynamics.

Dynamics	Key factors	Modulation	Effect on stem cell or iPSC behavior	References
Mito-fission	Drp1, Fis1	Downregulation of Drp1	Promote stem cell differentiation	[10, 16, 17]
			Lose stemness	[17, 18]
			Decrease reprogramming efficiency to iPSCs	[11, 19]
		Downregulation of Drp1/Fis1	Block apoptosis	[38–41]
		Upregulation of Drp1	Improve reprogramming efficiency to iPSCs	[20]
			Lose stemness	[21, 22]

Mito-fission	OPA1, Mfn 1/2	Downregulation of OPA1/Mfn1/2	Impair stem cell differentiation	[12, 42, 45]
			Promote neuron stem cell differentiation	[30]
			Impair iPSC differentiation	[44]
			Impair self-renewal	[30, 47]
			Improve reprogramming efficiency	[9]
		Upregulation of Mfn 2	Promote stem cell differentiation	[43]
			Induce iPSC differentiation	[44]
			Protect cell from apoptosis	[48, 49]

Mitophagy	Pink1, Parkin, Atg12, Atg3, Bnip3	Downregulation of Atg12/Atg3/Fis1/Pink1/Parkin	Impair self-renewal	[50–53]
			Decrease reprogramming efficiency to iPSCs	[51, 54]
		Downregulation of Atg12/Pink1	Promote stem cell differentiation	[50, 54]
		Downregulation of Atg3	Display abnormal differentiation	[51]
		Downregulation of Pink1	Impair neuron stem cell differentiation	[57]
		Downregulation of Pink1/Parkin/Bnip3	Lose the function of protecting the cell from apoptosis	[59–61]

Mito-biogenesis	PGC1*α*	Inhibition of biogenesis	Inhibit differentiation	[62]
		Inhibition or activation of biogenesis	Lose stemness	[64, 65]
		Inhibition of biogenesis	Cause cell death	[66]
		Activation of biogenesis	Promote stem cell differentiation	[63]

This table includes most key factors that are directly involved in mito-fission, mito-fusion, mitophagy, and mitochondrial biogenesis that are mentioned in this review. The effects of these key factors on stem cell behaviors are listed with the numbers of the references.

**Table 2:** A summary of pharmacological tools for mito-fission, mito-fusion, and mitochondrial biogenesis modulation and their reported effects.

Name	Function and mechanism	Physiological effect in nonstem cell	Physiological effect in stem cell
mDivi-1	Fission inhibitor: inhibit assembly of Drp1 and its GTPase activity	Prevent cell death	Prevent stem cell death; promote hiPSC differentiation
P110	Fission inhibitor: block Drp1/Fis1 interaction	Prevent stress- or injury-induced cell death	N/A
Dynasore	Fission inhibitor: noncompetitively inhibit the Drp1 GTPase activity	Protect cardiomyocyte from ischemia/reperfusion injury	N/A
M1	Fusion activator	Reduce cytochrome *c* release and protect rotenone-induced cell death	N/A
Leflunomide	Fusion activator: promote fusion by inhibition of pyrimidine synthesis	Protect cells from apoptosis	N/A
XCT790	Biogenesis inhibitor: inhibit ERR*α*-PGC-1 signaling pathway	N/A	Induce cancer stem cell death; induce cell cycle arrest
Azithromycin or doxycycline	Biogenesis inhibitor: inhibit mitochondrial protein translation	N/A	Induce cancer stem cell death

This table lists the reported effects of mito-fission inhibitors, mito-fusion activators, and mitochondrial biogenesis inhibitors in nonstem cells and stem cells. N/A denotes no study has been conducted.
